# Fintech, Bank Risk-Taking, and Risk-Warning for Commercial Banks in the Era of Digital Technology

**DOI:** 10.3389/fpsyg.2022.934053

**Published:** 2022-07-13

**Authors:** Gang Li, Ehsan Elahi, Liangliang Zhao

**Affiliations:** ^1^School of Economics, Shandong University of Technology, Zibo, China; ^2^Graduate School, Jungwon University, Cheongju, South Korea

**Keywords:** fintech, commercial bank, risk-taking, risk-warning, China

## Abstract

Fintech risks commercial banks in three ways, particularly operational efficiency, financial innovation, and risk management. Based on the data of 37 Chinese-listed commercial banks from 2011 to 2020, the study empirically analyzes the impact of fintech on bank risk-taking, and the intermediary effects of the three channels, such as operational efficiency, financial innovation, and risk management. The results show that fintech can effectively reduce the risk of banks. The results of heterogeneity analysis revealed that fintech strongly affects the risk-taking of state-owned banks but not obviously for rural commercial banks. Financial efficiency, financial innovation, and risk management indirectly affect the risk-taking of banks that contributed 8.51, 7.18, and 5.77%, respectively. We also constructed the commercial bank risk-warning index. Based on the quarterly data of banks from 2011 to 2020, we empirically tested the early warning effect of the bank risk-warning index. The results showed that when the signal month is set to 12 months, the bank risk-warning index can have a warning effect in this period.

## Introduction

In the era of the third technological revolution, digital technology, such as big data and cloud computing, is developing rapidly. Originated from the merging of finance and technology, fintech emerged as the times required dynamic integration. The emergence and development of financial technology have a significant impact on financial institutions, especially commercial banks.

Fintech has accelerated financial disintermediation, causing a large number of customers to leave commercial banks and turn to fintech companies. The development of fintech big data, cloud computing, and other technologies has virtually weakened the credit functions of traditional commercial banks, such as the influence of third-party payment models. The convenient and diverse third-party payment business bypasses bank operations and reduces the credit functions of commercial banks. Traditional commercial banks are affected by their own management culture and lack of experience and are slow to respond to new things such as financial technology. The development ideas have not changed because traditional commercial banks lack professional fintech talents, and the development of fintech enterprises has occupied the market share of traditional banking. Since the competition among commercial banks has increased, profits have declined.

Fintech has broken the limitations of time and space (Sun, 2021), enabling commercial banks to have more customers in a larger area. Online services and intelligent robots brought by financial technology have replaced offline labor, and costs are reduced. Fintech can enhance the innovative awareness of commercial banks, attach importance to innovative talents, and constantly innovate to create more new products and new services. Commercial banks actively cooperate with financial technology companies, which can be more accurately identified through technologies such as big data customers and improve the risk pricing capability of commercial banks.

In the process of creating challenges or providing opportunities for traditional commercial banks, financial technology will inevitably have risks, which will have a negative impact on the reliable and stable development of commercial banks. Since fintech has not changed the function and essence of its financial intermediary, it still has traditional financial risks and makes traditional financial risks more hidden. Moreover, fintech risks also include moral hazards and technical risks based on Internet software and hardware. The academic research in this field mainly focuses on the performance of commercial banks and the operational efficiency of commercial banks, and most of the research mainly analyzes the financial activities carried out by fintech enterprises. Limited studies have focused on the fundamental risk of fintech on commercial banks. Therefore, this study focuses on the estimation of risks of commercial banks due to fintech to provide strategies to reduce the risks.

## Literature Review

The risk-taking of financial technology for commercial banks is different from different perspectives. The following text will discuss how financial technology is responsible for the risk-taking of commercial banks from the perspectives of competition and cooperation.

When we focus on the perspective of competition, the development of fintech will bring challenges to the banking industry. In the asset business, Qiu et al. (2018) found that the continuous development of financial technology will lead to the development of interest rates in a market-oriented direction, which will undoubtedly change the liability structure of commercial banks, then the cost of liabilities of commercial banks will rise. To reduce losses caused by rising costs, commercial banks will invest in projects with higher risks and higher returns. Regarding debt, Berger and Houle (2019) found that in the continuous development of Internet technology, a large amount of funds does not need to pass through traditional financial institutions such as commercial banks as before and only needs to be transferred through the Internet platform. Although this efficient method is beneficial to the supply and demand of funds, it will undoubtedly have a certain impact on the important profitable business of traditional banks–the lending business. In the intermediary business, Nie and Liu (2021) analyzed that, with the development of financial technology, the convenient three-party payment business continues to squeeze intermediate business, such as the original water, electricity, gas agent payment, and agency insurance that were handled by commercial banks. Moreover, commercial banks also provide loan services to adopt green energy technology to reduce greenhouse gas emissions and climate change (Elahi et al., 2021a,b, 2022a,2022b).

When we focus on the perspective of cooperation, it will be beneficial to the development of commercial banking itself if fintech is actively integrated with commercial banks. In empirical research, Wu X. F. (2021) pointed out that the relationship between financial technology and commercial banks is not an alternative relationship but complementary. Financial technology can promote commercial banks to carry out products and services and other innovations to improve operational efficiency and reduce costs. Li et al. (2018) found that commercial banks can learn technical means such as the Internet, big data, and artificial intelligence by integrating with financial technology and using these technical means to obtain customers’ risk-taking levels and the changing laws of risk-taking levels, which is conducive to planning asset allocation that meets the specific financial needs of customers. Onay and Ozsoz (2013) found that, after the opening of the new business of online banking, customers’ deposit and loan behaviors in commercial banks increased significantly, and the commercial banks’ deposit and loan business continued to rise. The increase is reflected that the total assets of commercial banks rising, the return on assets rising, and the rate of nonperforming loans has fallen. Therefore, we know that the integration of commercial banks’ business and Internet technology has promoted the development of commercial banks themselves. Guo and Shen (2015) found that if commercial banks actively use Internet financial technology, management costs and bank risks can be reduced.

Previous studies have focused on financial risk-warnings using various types of regression techniques. For instance, the mainstream models are the probability regression functions (Probit and Logit) proposed by Frankel and Rose (1996), the cross-sectional regression model (STV) proposed by Sachs et al. (1996), the signal analysis method (KLR) proposed by Kaminsky and Zhang (1997), and the artificial neural network (ANN) model established by Mitra and Balaji (2010). Due to the rapid development of computer technology, a support vector machine (SVM) has become a new and effective risk-warning method in the field of artificial intelligence. The ANN has been used in various disciplines of social science (Elahi et al., 2019a,b). This method solves the problem that the dimension of the training samples is too high, and the data processing is difficult. Plenty of studies have confirmed that the SVM risk-warning effect is better. For example, Andrawis et al. (2010) found that SVM has a better classification function, and its classification accuracy is significantly improved compared to a back propagation (BP) neural network. Currently, there are relatively few studies on the risk-warning of financial technology, mainly focused on the risk early warning model of P2P online lending platforms. P2P refers to peer-to-peer network lending, and it is a type of Internet financial product.

## Theoretical Framework

### Fintech

#### Definition of Fintech

We can regard financial technology as a combination of finance and technology. The definition of fintech is not uniform in academia. According to the Financial Stability Board, fintech is financial innovation driven by the continuous development and advancement of technology (Ramlall, 2018). In addition, many scholars also conducted research and discussion on the definition of financial technology based on the existing definition and their understanding.

#### The Main Technology of Fintech

##### Artificial Intelligence

Artificial intelligence (AI) extends human intelligence to computer systems. In the financial field, due to the many manual links involved in the commercial banking business, diverse customers, coupled with unstructured financial data, complex business logic, and other factors. Artificial intelligence can be applied to all aspects of the financial industry with its advantages. Currently, the proportion of artificial intelligence in the investment of fintech enterprises is expanding and gradually increasing. It can be seen that the importance attached by financial technology companies to artificial intelligence also reflects the significant advantages of artificial intelligence. In the future, AI will occupy a larger market.

##### Big Data

Many processing methods have been used to capture the required data, manage the data, and process it into valuable information. In this way, the complex data are filtered and processed into a kind of data with great utilization value. Moreover, financial technology companies promote the innovation of financial products and services due to advanced technology, which allows more customers to choose. The consumption data of these customers is used by financial technology companies. After the collection of data by the financial technology companies, the banks can organize and analyze the data for personal credit investigation, credit extension, and risk control.

##### Cloud Computing

Cloud computing refers to the transfer of computations originally performed on local servers to the cloud for on-demand use. The method has low cost and high efficiency. Due to its unique advantages, many large network companies have joined the ranks of cloud computing, which has promoted the rapid development of cloud computing. In the financial field, commercial banks should also learn how financial technology companies use cloud computing to transfer computing to the cloud.

##### Blockchain

A blockchain is a distributed shared accounting mechanism that has various characteristics such as decentralization, immutability, and anonymity. This implies that it meets the business needs of the financial industry for data security, transaction authenticity, privacy, and confidentiality. In addition, many countries also strongly support the development of blockchain and have introduced many policies that are conducive to the development of blockchain.

### Risk-Taking

The commercial bank risk refers to the difference between the actual income obtained by a commercial bank after a period of operation and the income predicted by various methods. Due to a certain gap, the commercial banks cannot make normal profits and it causes losses in additional income.

Risk-taking refers to the fact that a company actively chooses to take risks to obtain high returns. Many studies have focused on the risk-taking of commercial banks. We emphasized the studies that focused on the risk-taking of commercial banks from the perspective of competition and innovation. Based on an empirical analysis of the issue from the perspective of cross-industry competition and horizontal competition constraints, Ying and Zhang (2020) reported that if the banks actively pursue profits, they will increase risks. Commercial banks will reduce risk-taking because of the government’s intervention. In general, the risk tolerance of commercial banks will be affected by two forces in different directions, and its ultimate risk tolerance will depend on the strength of the two opposite forces. Some scholars believe that financial innovation can improve the risk-taking ability of commercial banks, while others contradict this opinion. In recent years, many scholars believe that the impact of financial innovation on the risk-taking ability of commercial banks is bidirectional or even nonlinear. Chen et al. (2022) used the systematic GMM estimation method to judge the statements in the above literature. They concluded that there is a very obvious “inverted U”-shaped relationship between the degree of financial innovation and the risk-taking ability of commercial banks.

### Impact of Financial Technology on the Risk-Taking Mechanism of Commercial Banks

Information technology is a key factor driving the continuous development of banks and has significant technological spillover effects on the banking industry. According to the technology spillover theory, technology has external characteristics, and financial technology companies can empower commercial banks with their comparative advantages in information technology. Then, commercial banks adjust business thinking to upgrade their technology, transform their business, and improve the bank’s ability to deal with risks. On the one hand, the empowerment of financial technology can gain insight into the potential needs of users, achieve more accurate product pricing and more in-depth product development, and reduce the risk of product development failure. On the other hand, the development of financial technology can alleviate the problem of information asymmetry faced by commercial banks, improve the availability and accuracy of information when banks lend, increase information channels and sources, reduce information friction between banks and borrowers, and reduce loss of risk. However, the use of fintech by banks may also have the potential to increase their risk-taking, mainly due to technical risks and internal management risks. The development of financial technology is not long, and many banks have limited strength and lack core technical strength, which may lead to theft, leakage, and tampering of data and information. However, with the gradual improvement of the financial technology risk control system of China’s commercial banks and the increasing supervision of financial technology risks by the regulatory authorities, the advantages of China’s commercial banks’ development of financial technology outweigh the disadvantages. Based on the above analysis, we propose hypothesis 1 (H1).

H1: The improvement in the level of fintech can effectively reduce risk-taking.

When commercial banks and financial technology are integrated, the risks from financial technology are inevitably integrated. The risks of financial technology are transmitted through innovative financial products, customer experience, capital flow, and other channels and affect commercial banks’ risk-taking through operational efficiency, financial innovation, and risk-management effects.

#### Operational Efficiency

According to the theory of the technology spillover effect, the technology and technical talents of fintech spill over into the field of traditional commercial banks, promoting the development of traditional commercial banks. Fintech has technical spillovers from the demonstration effect, linkage effect, personnel flow effect, and competition effect, which will increase the operational efficiency of commercial banks and reduce the experience risk of commercial banks.

According to Schumpeter’s innovation theory, the development of financial technology can save or replace basic production factors such as labor, capital, and land to reduce the operating cost of commercial banks and narrow the gap between the actual and expected cost. The development of financial technology can also innovate business models and embed banking products and services into the online life scenarios of the public so that financial services can cover remote areas that are difficult to reach by physical outlets, open up new markets, and expand channels for banking business. Meanwhile, fintech can enhance customer acquisition capabilities, expand business scale, and directly increase the operating income of commercial banks, which is conducive to improving the revenue efficiency of commercial banks.

The development of financial technology is not only conducive to improving the cost efficiency of commercial banks but also to improving their revenue efficiency. When the cost efficiency and revenue efficiency of commercial banks are improved, their operating efficiency will improve, ultimately reducing their costs and risk-taking. Therefore, we propose hypothesis 2 (H2).

H2: The improvement in the level of banks’ financial technology can reduce the risk-taking of commercial banks by improving operational efficiency.

#### Financial Innovation

Fintech is essentially a technological innovation, and the most significant feature that distinguishes it from traditional finance is “technicization.” The integration of fintech companies and commercial banks based on digitization, automation, and intelligence provides commercial banks with a new service model. This change creates opportunities for commercial banks to carry out off-balance sheet business and promote diversified operations. Meanwhile, it greatly improves the business model of traditional commercial banks and enhances their level of financial innovation. With the advancement of technology, banks can not only improve the management efficiency of the entire internal capital market by financial innovation but also make it easier for other higher-yielding assets to be converted into money as a means of payment, which is very important for businesses that rely on the source of income from deposit and loan spreads. It is very attractive for banks. The improved level of banking financial innovation will help banking institutions to transfer risks and reduce their risk-taking. Gu and Zhou (2016) also found that the rising level of financial innovation in banks is beneficial to increasing the bank’s cumulative net income and reducing its willingness to provide risk capital. It can be seen that the use of financial technology by banks can improve their financial innovation level, increase their diversification, and reduce their risk-taking. Therefore, we propose hypothesis 3 (H3).

H3: The improvement in the level of banks’ fintech can reduce risk-taking by enhancing the level of financial innovation.

#### Risk Management

From the perspective of risk management, fintech, especially big data and technology supervision, will change the risk-management model of commercial banks and improve their risk-management capabilities, which will reduce the ultimate risk. Risk management is an important factor affecting the risk-taking of banks. Relying on financial technology, commercial banks can effectively enhance the effectiveness, accuracy, timeliness, and stability of risk management, especially in risk identification and risk assessment. First, in risk identification, traditional commercial banks have a single information acquisition channel. While relying on financial technology, traditional commercial banks can break through the limitations of time and space, expand the coverage of customers to the greatest extent, and diversify data dimensions. Then, banks can effectively solve the problems such as insufficient information and untimely updates. Second, in risk assessment, traditional commercial banks are limited by technology and compliance, and the utilization rate of external data is relatively low, coupled with the high proportion of manual review. It is difficult to accurately describe the “exposure level” of risks, while the application of artificial intelligence and big data can promote the intelligentization of bank risk assessment. For example, big data risk control models can conduct multidimensional analyses of risk-measurement indicators, which are helpful to capture the interaction effects between different variables and can more accurately describe the default characteristics of users, and then improve risk management and control capabilities. Based on the above discussion, we may propose hypothesis 4 (H4).

H4: The improvement in the level of banks’ fintech can reduce risk-taking by improving the risk-management capabilities.

## Materials and Methods

### Data Collection and Selection of Sample

From 2011 to 2020, data were collected from the Wind database, the annual reports of listed commercial banks, the website of the National Bureau of Statistics, and the website of China. There are 37 listed commercial banks (Table 1). Among them, five are state-owned commercial banks, eight are joint-stock commercial banks, 14 are city commercial banks, and 10 are rural commercial banks. The five state-owned commercial banks are the largest group of commercial banks in China. Moreover, eight joint-stock banks are the commercial banks established in accordance with the joint-stock system after the reform and opening in 1979. Similarly, 14 city commercial banks are regional commercial banks established in developed cities in China. In the last decade, 10 rural commercial banks are restructured from rural credit cooperatives.

**TABLE 1:** Selection of sample.

Types of bank	Quantity	Banks
State-Owned Commercial Banks	5	Industrial and Commercial Bank of China, China Construction Bank, Agricultural Bank of China, Bank of China, and Bank of Communications
Joint-stock Commercial Bank	8	China Merchants Bank, China CITIC Bank, Ping An Bank, Shanghai Pudong Development Bank, China Everbright Bank, Hua Xia Bank, Minsheng Bank, and China’s Industrial Bank
City Commercial Bank	14	Bank of Shanghai, Bank of Beijing, Bank of Ningbo, Bank of Nanjing, Bank of Guiyang, Bank of Suzhou, Bank of Zhengzhou, Bank of Qingdao, Bank of Hangzhou, Bank of Xi’an, Bank of Xiamen, Bank of Changsha, Bank of Chengdu, and Bank of Guiyang
Rural Commercial Bank	10	Jiangsu Suzhou Rural Commercial Bank, Qingdao Rural Commercial Bank, Jiangsu Zijin Rural Commercial Bank, Jiangsu Zhangjiagang Rural Commercial Bank, Chongqing Rural Commercial Bank, Shanghai Rural Commercial Bank, Jiangsu Jiangyin Rural Commercial Bank, Jiangsu Changshu Rural Commercial Bank, Wuxi Rural Commercial Bank, and Zhejiang Shaoxing Ruifeng Rural Commercial Bank

### Selection of Variables

#### Explained Variable

The explained variable is commercial banks’ risk-taking (*RISK*). In the previous studies, the variable has been used to measure commercial banks’ risk-taking with *z*-value, risk-weighted asset ratio, non-performing loan ratio, capital adequacy ratio, loan loss reserve ratio, capital asset ratio, equity-to-liability ratio, expected default probability, stock market volatility, and stock price volatility. According to the availability of data, we used the *z*-value (*Z*) to measure the overall risk level of commercial banks.


(1) $$\text{z}=\frac{R\,O\,A+C\,A\,R}{\sigma \,\left(R\,O\,A\right)}$$

where *ROA* is the return on assets, *CAR* is the capital-to-asset ratio (CAR = E/A), and σ(*ROA*) is the standard deviation of the return on assets. The *Z* value measures the overall stability of the banks. The changes in the *Z* value are consistent with the change in the stability of the banks. A larger *Z* value means stronger stability of banks. The change in the strength of stability is the opposite of the increase or decrease in risk. A bank with enhanced stability will reduce its risk, and *vice versa*. The *Z* value has its unique characteristics, showing a tail after the peak, so the logarithm of the *z* value must be taken during regression. There are special cases where the *z*-value is zero, and *log* (1+Z) is used instead of *log* (Z). In addition, we used the nonperforming loan ratio (*NPL*) to replace the *z*-value for robustness analysis.

#### Main Explanatory Variables

In the literature, there are many indicators to measure the degree of financial technology development. For example, Qiu et al. (2018) used China’s Digital Financial Inclusion Index. Guo and Shen (2015) used the “text mining method” to build their Internet financial index. Zhu and Hua (2018) used the “Internet Finance Theme Index” compiled by the Shanghai Stock Exchange to replace the development scale of Internet finance.

By analyzing the measurement methods above, we used the “text mining method” of Guo and Shen (2015) to construct our fintech index and used the method of Wu X. F. (2021) to measure the degree of independent use of financial technology by banks, namely, the fintech level of commercial banks. Specific steps can be written as follows:

The first step is to establish an initial lexicon of fintech based on the functions of fintech in commercial bank applications. At the same time, starting from the format of business, we divided financial technology into four dimensions, such as payment and settlement, business development, investment management, and resource allocation.

The second step is to match each keyword with the bank name, search for the bank name and keyword in Baidu Information, and then use Python web crawler technology to crawl all the news search results of each bank from 2011 to 2020. To ensure the accuracy of search results, we used double quotation marks to lock keyword groups when searching to filter out irrelevant or wrong information.

The third step is to filter effective keywords. In particular, the word frequency is standardized, and then the Pearson correlation analysis method was used to calculate the correlation coefficient between the word frequency and the annual average value of the nonperforming loan ratio of commercial banks. Following Larson and Farber (2011), the critical value of weak correlation is set at 0.3. Finally, a total of 15 keywords were reserved, such as peer-to-peer transmission, digital currency, value transfer network, foreign exchange wholesale, third-party payment, online payment, online financing, Internet financial management, Internet of Things, artificial intelligence, blockchain, large-scale data, cloud computing, and digital exchange platform.

The fourth step is to synthesize the annual fintech index of each bank (*FIN*). The number of news search results for all keywords at the annual level of each bank is summed up to obtain the annual total news volume of the sample bank, and then logarithmically processed to serve as an indicator to measure the bank’s annual level of fintech use. It should be pointed out that to accurately identify the business orientation of the commercial bank fintech index, we decomposed the fintech index of each bank into four dimensions, namely, payment settlement (*PAYS*), business development (*BUS*), investment management (*INM*), and resource allocation (*RESA*), and measured the sub-indicators of each dimension.

#### Mediating Variables

Fintech mainly affects the overall risk level of commercial banks through three channels such as operational efficiency, financial innovation, and risk management. To count the impact of financial technology on these aspects, we transformed them into measurable indicators. We used the cost-to-income ratio (*CIR*), noninterest income ratio (*NII*), and loan impairment to total loan ratio (*ILR*) to represent operating efficiency, financial innovation, and risk management to analyze the intermediary effect.

#### Control Variables

To analyze the changes in the explained variables and minimize the multicollinearity problem more efficiently, we added some control variables, including macro-level and microlevel. The value of variance inflation factor (VIF) for multicollinearity is around 5, which reveals the non-existence of the multicollinearity.

##### Macroeconomic Variables

###### Economic Development (GGDP)

The higher the GDP growth rate, the better the economic development, which affects the business and operations of commercial banks. Generally, there is an inverse relationship between the GDP growth rate and the risk-taking of commercial banks.

###### Inflation

There are three possibilities for the impact of inflation (INF) on bank risk. First, inflation will increase bank costs, which is adverse for banks. Second, the central bank’s currency is over-issued, and inflation is beneficial for the bank, which is the debtors. Third, when the economy is prosperous, inflation will make the country use tight monetary policy to curb inflation, which is adverse for the banking system.

###### Monetary Policy (M_2_)

We used the growth rate of the broad money supply to represent monetary policy. The lower the cost of financing by enterprise, the looser the monetary policy. The larger-scale credit business of commercial banks and the relaxation of credit standards are detrimental to credit risk management.

##### Variables of Bank Characteristics

###### Operating Scale

Logarithm of total assets (LNTA) belongs to the operating scale of a bank, which is generally expressed by the bank’s total assets or total income. According to the theory of economy of scale and scope, the expansion of scale can reduce the credit risk faced by banks. We have taken the bank’s asset size as one of the control variables and taken the logarithm of the asset size.

###### Capital Structure

We used the owner’s equity ratio (*OER*) to measure the bank’s capital structure. The *OER* is equal to the ratio of equity capital to total assets. The higher the *OER*, the less debt the bank has, the stronger the repayment ability and the stable capital structure. In addition, we also used control of the micro-characteristics of commercial banks, such as return on assets (*ROA*), net interest margin (*NIM*), and loan-to-deposit ratio (*LOD*).

### Analytical Framework

To verify the impact of commercial bank financial technology development on its risk-taking, we used the given regression function.


(2) $$R\,I\,S\,K_{i\,t}=\alpha_{0}+\alpha_{1}\,F\,I\,N_{i\,t}+\beta \,c\,o\,n\,t\,r\,o\,l\,s_{i\,t}+\mu_{i}+\lambda_{t}+\varepsilon_{i\,t}$$

where *RISK* is the explained variable and represents the bank’s risk-taking, the main explanatory variable (*FIN)* represents the extent of the bank’s financial technology, *controls*_*it*_ is a set of control variables, μ_*i*_ represents the individual fixed effect, the λ_*t*_ represents time fixed effect, and the ε_*it*_ is the random error, which is assumed to be normally distributed at zero mean value and constant variance (Elahi et al., 2017, 2018a,2018b,2019c,^2020^) and *i* = 1, 2, …, 37, and *t* = 2011, 2012, …,2020.

In addition, we used an intermediary effect model to examine whether fintech affects risk-taking through intermediary indicators. The regression functions can be written as follows:


(3) $${\text{Med}}_{i\,t}=C{+}_{2}+\alpha FIN_{i\,t}+\beta contorls_{i\,t}+\mu_{i}+\lambda_{t}+\varepsilon_{i\,t}$$

(4) $${\text{z}}_{i\,t}=C+\alpha_{3}\,x_{i\,t}+\delta \,M\,e\,d_{i\,t}+\beta \,c\,o\,n\,t\,o\,r\,l\,s_{i\,t}+\mu_{i}+\lambda_{t}+\varepsilon_{i\,t}$$

The intermediary variables (_*Med_it*_) are operational efficiency, financial innovation, and risk management.

### Construction of a Risk-Warning Model for Commercial Banks

The KLR refers to Kaminsky, Lizondo, and Reinhart because it was introduced by Kaminsky, Lizondo, and Reinhart in 1998. The KLR model is a signal analysis method that is used to study bank risk and financial crisis early warning. The main idea of the KLR model is to build an indicator system and combine these indicators to determine the threshold range. When the predicted data exceed the threshold range, a danger warning signal will be issued. The higher the indicator value, the greater the risk corresponding to the indicator. The prediction time window of the KLR model was set to 12 months and decomposed the signal into four categories. Suppose “A” represents the number of correct signal months, “B” is the number of false signal months, “C” is the number of months that should have been signaled but were not signaled, and “D” is the number of months that should not have been signaled but were not signaled. If the crisis signal occurs within the 12-month range, the signal is valid, and if the crisis signal occurs after 12 months, the signal is invalid.

According to the assumption, the ideal model of the early warning model is that “A” and “D” are greater than 0, and “B” and “C” are equal to 0. Based on the actual situation, if the ideal state cannot be achieved, Kaminsky and Zhang (1997) used the method of minimizing the noise-signal ratio to determine the indicator threshold. A/(A+C) represents the ratio of valid signals; B/(B+D) represents the ratio of invalid signals; and [B/(B+D)]/[A/(A+C)] represents the signal-noise ratio to the effective signal. The indicator is abbreviated as NSR, and the case with the smallest indicator is the best threshold. There are n indicators. *X*_*it*_ represents the value of indicator *i* in time period *t*, and *S*_*it*_ represents whether there is a danger signal for indicator *i* in time period *t*. *S_*it*_* = 1 means that the indicator *i* sends a danger signal in the *t* time period, and *S_*it*_* = 0 shows that the indicator *i* does not send out a danger signal in the *t* time period. Following Bisceglia and Scigliuto (2016), we constructed the bank risk index (*BRI*).


(5) $$B\,R\,I_{t}=w_{L\,d\,r}\,\left(\frac{L\,d\,r_{t}-L\,d\,r_{t-1}}{L\,d\,r_{t-1}}\right)+w_{R\,i\,r}\,\left(R\,i\,r_{t}-R\,i\,r_{t-1}\right)+w_{M}\,\left(\frac{M_{t}-M_{t-1}}{M_{t-1}}\right)$$

where *Ldr*_*t*_, *Rir*_*t*_, and *M*_*t*_ represent the loan-to-deposit ratio, real interest rate, and money supply at time *t* (or time period), respectively. *W*_*Ldr*_, *W*_*Rir*_, and *W*_*M*_ are the weights of the deposit-loan ratio, the real interest rate, and the money supply, respectively. The weights are determined by the standard deviation that can be calculated using a given function.


(6) $$w_{k}=\frac{1}{S\,t\,d_{k}}/\left(\frac{1}{S\,t\,d_{L\,d\,r}}+\frac{1}{S\,t\,d_{R\,i\,r}}+\frac{1}{S\,t\,d_{M}}\right)$$

The criteria for the early warning can be written as follows:


(7) $$W\,a\,r\,n\,i\,n\,g=\bar{B\,R\,I}+\sigma_{t}$$

If $B\,R\,I_{t}>\bar{B\,R\,I}+\sigma_{t}$, then the bank has systemic risk at time *t*. If $B\,R\,I_{t}<\bar{B\,R\,I}+\sigma_{t}$, then the systemic risk of the bank at the time *t* is small.

The bank risk-warning index can be written as follows:


(8) $$R\,W\,I=\frac{\sum_{i}\left(\frac{S_{i\,t}}{N\,S\,R_{i}}\right)}{\sum_{i}\left(\frac{1}{N\,S\,R_{i}}\right)}$$

where *NSR*_*i*_ represents the noise-to-signal ratio corresponding to the indicator *i*. The calculation formula is [B/(B+D)]/[A/(A+C)], and *Si*_*t*_ represents whether the indicator *i* has a danger signal in the *t* time period. *S_*it*_* = 1 means that the indicator *i* sends a danger signal in the *t* time period. When *S_*it*_* = 0, the indicator *i* does not send a danger signal in the *t* time period.

## Results and Discussion

### Descriptive Statics

The descriptive statistics are given in Table 2. It is found that the *Z* value of the explained variable (RISK) is distributed between -0.47 and 2.27, with a mean of 1.58 and a standard deviation of 0.87. It shows that during the statistical period, partial risks found in some years, but the overall Chinese banks, are robust with less change. Similar results were found in previous studies (Chen et al., 2018; Wang et al., 2018). The average value of FIN is 0.53, with a standard deviation of 0.37 and a distribution interval between 0 and 0.78. It shows that the financial technology of Chinese banks has been steadily improved with less fluctuation (Jia and Winseck, 2018; Yao et al., 2018). Although overall changes in other variables are stable, they fluctuate slightly in the past 10 years.

**TABLE 2:** Descriptive statistics of variables.

Types	Variables	Mean	Standard deviation	Minimum	Maximum
Explained variable (*RISK*)	*Z*	1.58	0.87	–0.47	2.27
	*NPL*	1.45	1.03	0.00	11.84
Explanatory variables	*FIN*	0.53	0.37	0.00	0.78
Explanatory Variable Decomposition Indicators	*PAYS*	0.71	0.41	0.00	0.95
	*BUS*	0.58	0.39	0.00	0.87
	*INM*	0.41	0.19	0.00	0.64
	*RESA*	0.07	0.11	0.00	0.16
Mediating variable	*CIR*	39.45	15.03	27.33	58.17
	*NII*	19.33	15.09	0.52	62.37
	*ILR*	4.11	1.12	2.77	6.87
Control variable	*GGDP*	7.05	1.41	2.30	17.22
	*INF*	1.96	0.81	1.80	2.08
	*M2*	11.17	24.80	8.17	13.28
	*LNTA*	14.45	1.40	10.25	18.55
	*OER*	6.75	1.68	1.63	23.54
	*ROA*	1.05	0.36	0.02	4.03
	*NIM*	2.63	0.84	0.09	7.09
	*LOD*	66.18	18.94	24.37	109.77

### Impact of Fintech on Commercial Banks’ Risk-Taking

We used a fixed-effect model to analyze the impact of fintech on commercial banks’ risk-taking (Table 3). It is found that both the full sample and different types of banks, fintech are significantly positively correlated with bank risk. The explained variable of bank risk is the *z*-value, which indicates the stability of the bank. Therefore, fintech can effectively improve the operational stability of the bank and reduce bank risk. The results verified hypothesis 1. The result of heterogeneity analysis revealed that state-owned banks are the most effective, and rural commercial banks are the worst effects in the risk prevention effect of financial technology on banks. State-owned banks have strong funds, obvious talent reserves, and technical advantages. Meanwhile, state-owned banks have strong research and development capabilities for key technologies related to financial technology, start technology earlier, and invest on a larger scale with more mature applications and devolvement. Moreover, state-owned banks’ business strategies are more conservative, with relatively rich experience in risk management and more prudent decision-making behavior. Therefore, when using financial technology, state-owned banks have the most obvious risk prevention effect and higher robustness. For rural commercial banks, due to their relatively weak R&D and innovation capabilities, financial technology research and development relies on third-party technology companies. Although the application of financial technology is conducive to reducing bank risks, the risk prevention effect is the least obvious.

**TABLE 3:** The impact of fintech on commercial banks’ risk-taking.

Variables	(1)	(2)	(3)	(4)	(5)
	Full sample	State banks	Joint-stock banks	City commercial banks	Rural commercial banks
*FIN*	2.187*** (0.339)	2.770*** (0.667)	2.509* (0.739)	2.024* (0.470)	1.659*** (0.639)
*GGDP*	1.538* (0.393)	1.632*** (0.549)	1.733* (0.133)	1.078* (0.690)	1.160*** (0.2 79)
*INF*	–1.380* (0.187)	–1.205*** (0.174)	–1.176*** (0.298)	–1.747 (0.296)	–1.265*** (0.184)
*M2*	0.290*** (0.164)	0.342** (0.088)	0.304*** (0.140)	0.293*** (0.040)	0.292*** (0.027)
*LNTA*	0.098* (0.030)	0.151 (0.049)	0.166*** (0.081)	0.091* (0.093)	0.081 (0.077)
*OER*	1.787* (0.831)	1.772* (0.728)	1.338* (0.873)	1.239* (0.741)	2.072** (0.729)
*ROA*	0.396* (0.294)	–1.374 (0.829)	1.098 (0.763)	1.884* (0.931)	1.038*** (0.770)
*NIM*	1.884* (0.986)	0.942*** (0.790)	1.306* (0.501)	2.554*** (1.410)	2.183** (0.873)
*LOD*	0.322* (0.115)	0.295* (0.136)	0.380*** (0.108)	0.335 (0.174)	0.366*** (0.110)
Time effect	Control	Control	Control	Control	Control
Individual effect	Control	Control	Control	Control	Control
Number of samples	361	50	80	136	95
*R* ^2^	0.621	0.703	0.694	0.504	0.448

***, **, and * represent the level of significance at p < 0.01, p < 0.05, and p < 0.1, respectively. Standard errors are given in parentheses.

Furthermore, we examined the impact of financial technology indicators on commercial banks’ risk-taking (Table 4). For the full sample data, the three dimensions, namely, payment and settlement, business development, and investment management, have a significant positive impact on the stability of commercial banks. It indicates that for the full sample, financial technology plays an important role in the field of payment and settlement, business development, and investment management applications. Fintech will significantly improve the operational stability of commercial banks and reduce bank risks. In terms of different banks, the two dimensions of payment settlement and investment management have significant positive effects on state-owned banks, joint-stock banks, city commercial banks, and rural commercial banks. The dimension of business development has a significant positive effect on state-owned banks, joint-stock banks, and rural commercial banks. The dimension of resource allocation has a significant positive effect on state-owned banks and joint-stock banks. For state-owned and joint-stock banks, the application of financial technology in the field of payment and settlement, business development, investment management, and resource allocation can help to improve their stability and reduce bank risk-taking. For urban commercial banks, the application of fintech only in the field of payment and settlement and investment management will significantly improve the stability of their banks and reduce bank risk-taking. Similarly, for rural commercial banks, the application of fintech in the field of payment and settlement, business development, and investment management reduces bank risk-taking.

**TABLE 4:** The impact of various decomposition indicators of fintech on the banks’ risk-taking.

Variables	(1)	(2)	(3)	(4)	(5)
	Full sample	State banks	Joint-stock banks	City commercial banks	Rural commercial banks
PAYS	1.815** (0.821)	2.196*** (0.882)	1.947* (0.866)	1.438** (0.718)	1.077* (0.636)
BUS	1.763*** (0.708)	1.497** (0.684)	1.978** (0.831)	1.240 (0.575)	1.117** (0.567)
INM	2.026** (1.054)	2.457** (1.238)	1.839* (0.937)	1.719** (0.849)	1.600* (0.694)
RESA	2.308 (1.556)	2.844* (1.607)	2.235* (1.284)	2.011 (0.964)	1.862 (0.878)
Control variable	Control	Control	Control	Control	Control
Time effect	Control	Control	Control	Control	Control
Individual effect	Control	Control	Control	Control	Control
Number of samples	361	50	80	136	95
*R* ^2^	0.586	0.611	0.657	0.478	0.410

***, **, and * represent the level of significance at p < 0.01, p < 0.05, and p < 0.1, respectively. Standard errors are given in parentheses.

### Analysis of Mediating Effect

We used the mediation effect model to analyze the impact of fintech on banks’ risk-taking. It should be pointed out that the mediation effect analysis model is for the full sample data. Column 1 of Table 5 showed the results of basic regression for the total effect of financial technology on the risk-taking of commercial banks. While columns 2, 3, and 4 represent the regression results for financial technology (*FIN*) on the cost-income ratio (*CIR*), non-interest income ratio (*NII*), and loan impairment to total loan ratio (*ILR*), it represents the intermediary effect or indirect effect of financial technology on commercial banks’ risk-taking through operational efficiency, financial innovation, and risk management. Column 5 is the regression result of financial technology, cost-to-income ratio, noninterest income ratio, loan impairment to total loan ratio combined on the risk-taking of commercial banks, representing the direct effect of financial technology on commercial banks’ risk-taking, where the coefficient of financial technology represents the direct effect of the impact of financial technology on banks’ risk-taking.

**TABLE 5:** Impact of fintech on intermediary variables.

Variables	(1)	(2)	(3)	(4)	(5)
	RISK	CIR	NII	ILR	RISK
FIN	2.187*** (0.339)	1.208*** (0.711)	1.441*** (0.413)	1.752*** (0.308)	1.262*** (0.657)
CIR	—	—	—	—	0.154** (0.127)
NII	—	—	—	—	0.109** (0.096)
ILR	—	—	—	—	0.072* (0.008)
Control variable	Control	Control	Control	Control	Control
Time effect	Control	Control	Control	Control	Control
Individual effect	Control	Control	Control	Control	Control
Number of samples	361	361	361	361	361
*R* ^2^	0.498	0.511	0.553	0.561	0.620

***, **, and * represent the level of significance at p < 0.01, p < 0.05, and p < 0.1, respectively. Standard errors are given in parentheses.

The results of Table 5 confirmed that the estimated coefficients of the financial technology level of commercial banks (*FIN*) to the cost-to-income ratio (*CIR*), non-interest income ratio (*NII*), and loan impairment to total loan ratio (*ILR*) are all significantly positive. It indicates that the improvement of the level of financial technology will significantly improve their financial efficiency, financial innovation level, and risk management and control ability of commercial banks (Cherotich et al., 2015; Chen et al., 2017). When main explanatory variables are added to the intermediary effect model, the estimated coefficient of commercial bank fintech remains significant and positive. Moreover, the intermediary variables’ cost-income ratio (*CIR*), non-interest income ratio (*NII*), and loan impairment to total loan ratio (*ILR*) are also significant and positive. The results showed that financial efficiency, financial innovation, and risk management play an intermediary role in the impact of commercial banks’ financial technology level on their risk reduction. In the contribution of financial technology level to commercial banks’ risk-taking, the total effect is 2.187, the direct effect is 1.262, and the proportion of direct effect is 57.70%. The intermediary effects of financial technology in reducing its risk-taking through financial efficiency, financial innovation, and risk management account for 8.51, 7.18, and 5.77% of the total effect, respectively. It reveals that the three channels of improving financial efficiency, financial innovation, and risk management contribute 8.51, 7.18, and 5.77% to explain the improvement of banks’ fintech level and thus reduce their risk-taking. The empirical results effectively identified the transmission mechanism of financial technology through three paths of financial efficiency, financial innovation, and risk management, and then affected banks’ risk-taking. These findings satisfy hypotheses 2, 3, and 4.

### Test of Robustness

Following Zhang et al. (2016), we used the method of replacing the explained variables to test the robustness, specifically replacing the *Z* value with the nonperforming loan ratio (*NPL*) as a proxy for the risk-taking results of commercial banks. The robustness test results are shown in Table 6. It can be seen that the impact of fintech on the risk-taking results of commercial banks is still significant because the nonperforming loan ratio is a negative indicator, and the fintech coefficient is significantly negative. It indicates that fintech has reduced the risk-taking level of commercial banks. Moreover, it is confirmed that the basic test is robust.

**TABLE 6:** Robustness test.

Variables	(1)	(2)	(3)	(4)	(5)
	Full sample	State banks	Joint-stock banks	City commercial banks	Rural commercial banks
FIN	–0.017** (0.9206)	–0.018*** (0.7149)	–0.022* (0.8033)	–0.014* (0.7842)	–0.010* (0.6639)
Control variable	Control	Control	Control	Control	Control
Time effect	Control	Control	Control	Control	Control
Individual effect	Control	Control	Control	Control	Control
Number of samples	361	50	80	136	95
R^2^	0.329	0.352	0.347	0.294	0.271

***, **, and * represent the level of significance at p < 0.01, p < 0.05, and p < 0.1, respectively. Standard errors are given in parentheses.

### Analysis of Early Warning Mechanism

We used the KLR model to analyze the risk warning of commercial banks. The early warning indicators were constructed at the macro-level and microlevel. In particular, 10 early warning indicators were selected, and weights were given to the indicators. The KLR model was first proposed by Kaminsky and was mainly used for currency crisis research. Then, many people put it into early warning analysis of the financial crisis, early warning analysis of futures crisis, and early warning analysis of stock market systemic risk.

#### Construction of Risk-Warning Indicator System for Commercial Banks

##### Microlevel Indicators

At the microlevel, bank risk is closely related to the bank itself. The higher the risk of an individual business, the greater the vulnerability and the higher the probability of systemic risk. At the same time, the empirical results in the third part of the article showed that the application of financial technology can effectively reduce the risk level of banks. Therefore, we also incorporated financial technology into the risk-warning indicator system of commercial banks. Finally, in the selection of microlevel indicators, we determined five secondary indicators such as capital adequacy ratio, provision ratio, liquidity ratio, nonperforming loan ratio, and financial technology level.

##### Macro-Level Indicators

At the macro level, the level of economic development will affect the operation of banks. Whether it is pro-cyclical or countercyclical, the macroeconomy will affect the development of banks. The deterioration of the macroeconomy will be transmitted to the banks in various ways, such as the deterioration of the real economy, the failure of many business customers, and the inability to repay bank loans, resulting in credit risks. Therefore, in the selection of macro indicators, five secondary indicators are determined, such as the CPI growth rate, GDP growth rate, interest rate, exchange rate, and banking prosperity index (Table 7).

**TABLE 7:** Indicator system of commercial bank’s risk-warning.

Risk-warning indicator	Primary indicators	Secondary indicators
	Micro Indicators	Capital Adequacy Ratio
		Provision Ratio
		Liquidity Ratio
		Non-performing Loan Ratio
		Fintech Index
	Macro Indicators	CPI Growth Rate
		GDP Growth Rate
		Interest Rate
		Exchange Rate
		Banking Prosperity Index

### Analysis of a Risk-Warning Model of Commercial Banks

In this section, we selected the sample of the fourth part, the quarterly data from the first quarter of 2011 to the fourth quarter of 2020, a total of 40 time periods. In this study, we used ten indicators, particularly capital adequacy ratio, provision ratio, liquidity ratio, nonperforming loan ratio, financial technology level, CPI growth rate, GDP growth rate, interest rate, exchange rate, and banking prosperity index. We substituted the data into equation 4 to calculate the risk index of each bank.

#### Estimation of Indicator Warning Threshold

##### Correlation Analysis Between Each Index and Bank Risk Index

If an indicator is positively correlated with the BRI, the larger the indicator value, the higher the probability of bank systemic risk. If the indicator is negatively correlated with the BRI, the smaller the indicator value, the higher the probability of bank systemic risk. The capital adequacy ratio is negatively correlated with the BRI. The provision ratio is the ratio of provision for bad and doubtful debts, which is positively related to the bank’s risk index. The liquidity ratio reflects the asset liquidity of commercial banks and is negatively correlated with the BRI. The nonperforming loan ratio reflects the bank’s nonperforming loan situation and is positively correlated with the bank’s risk index. The level of fintech is negatively correlated with the BRI. The more severe the inflation, the greater the possibility of a financial crisis, so the CPI growth rate is positively correlated with the BRI. The economic growth is stable and the probability of a financial crisis is small; therefore, the GDP growth rate is negatively correlated with the BRI. Both interest rates and exchange rates are positively correlated with the BRI. The banking sentiment index is negatively correlated with the banking risk index. Previous studies have shown similar findings (Bordo et al., 2016; Tan and Floros, 2018).

##### Threshold and Optimal Noise Signal Ratio

Using the KLR method for crisis early warning analysis, it is necessary to clarify the thresholds of various indicators. We have taken the noise-to-signal ratio, which is the minimum NSR. The threshold and optimal NSR of each index are given in Table 8.

**TABLE 8:** Thresholds of each indicator and optimal noise signal ratio.

Indicators	Capital adequacy ratio	Provision ratio	Liquidity ratio	Non- performing loan ratio	Fintech index	CPI growth rate	GDP growth rate	Interest rate	Exchange rate	Banking prosperity index
Thresholds	11.52%	2.18	48.04%	0.97%	0.88	3.96%	9.54%	3.57%	6.38	88.14
Optimal noise signal ratio	0.43	0.57	0.17	0.56	0.24	0.05	0.20	0.09	0.31	0.73

If the indicator calculation result is within the threshold range, the system will not issue a risk warning signal. If the indicator calculation result exceeds the threshold range, the system will issue a risk warning signal. At the same time, it is also necessary to pay attention to the correlation analysis of indicators. If there is a positive relationship between the indicators and the crisis, an early warning signal will be issued when the indicators are higher than the threshold range. If there is a negative relationship between the indicator and the crisis, there will be no warning signal below the threshold range. It is also necessary to pay attention to the optimal noise signal ratio. The smaller the calculation result, the stronger the early warning ability of the corresponding indicator. Generally, the indicators with the optimal noise signal ratio higher than 1 can be directly eliminated, their reference value is not obvious, and the early warning ability is limited. For example, the optimal noise signal of the two indicators of banking climate index and nonperforming loan ratio is relatively large, indicating that the two indicators have low early warning ability. While the optimal noise signal of the CPI growth rate is relatively small, the indicator has a high warning ability. The contribution of the level of early warning ability to the final comprehensive early warning index of systemic risk is reflected in the weight of each index.

#### Test of Early Warning Effect

**FIGURE 1:**
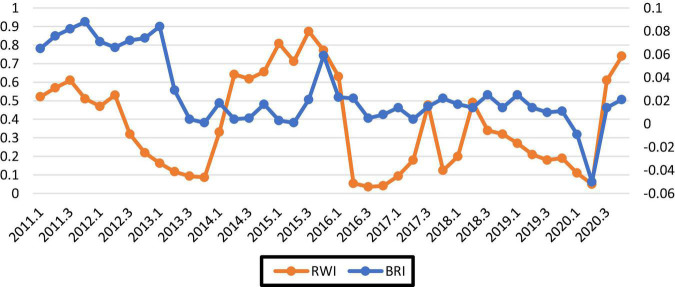
Bank’s overall risk index and risk-warning index.

According to the determined threshold range, the release of crisis signals is determined by the indicators, and the relevant values are substituted into equation 7 to calculate the bank’s risk early warning index (*RWI*). According to the sample and time selection, we calculated the *BRI* and bank risk-warning index (*RWI*) of 37 listed commercial banks from the first quarter of 2011 to the fourth quarter of 2020. By comparing the *BRI*_*t*_ and *RWI*_*t*_ of 37 banks, the risk-warning ability of each bank can be observed. Our early warning effect test focused on measuring the overall risk situation and early warning capability of the banks. The calculation results are shown in Figure 1. Comparing the trend of the BRI and the bank risk-warning index, it can be seen that if the signal month is set to 12 months, the bank risk-warning index can play a warning effect in this cycle. Observing the changing trend of the indicators BRIt and RWIt, the risk situation can be predicted. In fact, in addition to the strong predictive ability of the overall risk early warning system, the individual risk early warning index for each bank can also well predict the risk situation of each bank.

In the process of building the bank’s risk-warning index, it is found that the early warning capabilities of each index are different. Indicator correlation analysis can clarify the direction of macro-prudential management. Particularly, it effectively reduces the nonperforming loan ratio and CPI growth rate, comprehensively increases the relative proportion of liquidity, forms a sound early warning system structure, controls the capital periodicity of the adequacy ratio, ensures the stability of bank deposit interest rates, reduces the bank provision rate, and improves banks’ fintech degree. Through these concerns, the occurrence of systemic risk can be reduced.

## Conclusion and Policy Implications

### Conclusion

In this study, we estimated the impact of financial technology on commercial banks’ risk-taking and identified the transmission path through which financial technology affects commercial banks’ risks. The fixed-effect model is used to analyze the impact of fintech on commercial banks’ risk-taking. The results showed that both the full sample and all types of bank financial technology are significant and positively correlated with banks’ risk-taking. Fintech can effectively reduce bank risk. The results of heterogeneity analysis revealed that state-owned banks have the highest risk prevention effect on banks, and rural commercial banks have a relatively insufficient effect. We also examined the impact of financial technology indicators on commercial banks’ risk-taking and found that the impact of financial technology indicators on commercial banks’ risk-taking is slightly different.

Based on the full sample data, the mediation effect model is used to analyze the impact of financial technology on various channels of bank risk. The results showed that the estimated coefficients of commercial banks’ financial technology on cost-to-income ratio, noninterest income ratio, loan impairment, and total loan ratio are all significant and positive. It indicated that the improvement of financial technology significantly improved the financial efficiency and financial innovation level and risk management capabilities. Furthermore, the results confirmed that financial efficiency, financial innovation, and risk management generated an intermediary effect for the impact of commercial banks’ financial technology on risk reduction. To explain the improvement of banks’ fintech, it is found that the three channels of financial efficiency, financial innovation, and risk management contributed 8.51%, 7.18%, and 5.77%, respectively, to the reduction of risk-taking.

We also constructed the commercial BRI and the commercial bank risk early warning index (RWI). The warming effect of the bank risk early warning index based on the data from China’s banks has been found. The results showed that when the signal month is set to 12 months, the bank risk early warning index can play a warning effect in this period. In the process of building the bank risk early warning index, it is found that the early warning capabilities of each index are different.

### Policy Implications

Commercial banks should use fintech to expand their customers and reject the “2 and 8 rule” as before. Although the number of tail customers is small, they require individualization and differentiation. If banks meet their individualized and differentiated needs, a huge market will be formed. For example, for typical long-tail customers such as small enterprises, if banks can launch consumer financial services that meet their needs, they can master more information on long-tail customers. By mining the value of the entire chain and exploring new profit channels, banks can reduce risks. Meanwhile, commercial banks cannot ignore the original customer groups.

We also need to guard against the risks posed by fintech. Fintech is also a double-edged sword for banks. While fintech promotes the transformation and upgrading of banks, it also brings certain risks. Fintech innovation has made financial risks more hidden and concealed in every corner of the financial industry. Once the risk is exposed, the systemic risk will be triggered due to the contagion of the risk. And, not only the banks will be hurt, but the entire financial system will be hit hard. Therefore, when the technologies are used, comprehensive risk prevention and control must be done.

Banks should also use effective risk early warning mechanisms and technologies. Although fintech brings benefits to banks, it also brings risks. A useful risk early warning system can identify financial risks in advance. Commercial banks should actively develop appropriate risk early warning technologies, test the early warning capabilities of these technologies, and use these warning indicators to monitor their business, which can minimize the impact and loss caused by risks.

## Data Availability Statement

The raw data supporting the conclusions of this article will be made available by the authors, without undue reservation.

## Author Contributions

GL and LZ were responsible for the data collection, arrangement of relevant literature, and data analysis. EE made comments on the choice of the article, helped in writing the original draft of the manuscript, and revised the manuscript according to the comments of reviewers. All authors contributed to the article and approved the submitted version.

## Conflict of Interest

The authors declare that the research was conducted in the absence of any commercial or financial relationships that could be construed as a potential conflict of interest.

## Publisher’s Note

All claims expressed in this article are solely those of the authors and do not necessarily represent those of their affiliated organizations, or those of the publisher, the editors and the reviewers. Any product that may be evaluated in this article, or claim that may be made by its manufacturer, is not guaranteed or endorsed by the publisher.
